# Imatinib reduces non-alcoholic fatty liver disease in obese mice by targeting inflammatory and lipogenic pathways in macrophages and liver

**DOI:** 10.1038/s41598-018-32853-w

**Published:** 2018-10-17

**Authors:** Shefaa AlAsfoor, Theresa V. Rohm, Angela J. T. Bosch, Thomas Dervos, Diego Calabrese, Matthias S. Matter, Achim Weber, Claudia Cavelti-Weder

**Affiliations:** 1 Clinic of Endocrinology, Diabetes and Metabolism, University Hospital Basel, Basel, Switzerland; 2 Department of Biomedicine, University of Basel and University Hospital Basel, Basel, Switzerland; 3 Institute of Pathology, University Hospital of Basel, Basel, Switzerland; 4 Department of Pathology and Molecular Pathology, University and University Hospital of Zurich, Zurich, Switzerland

## Abstract

Macrophages have been recognized as key players in non-alcoholic fatty liver disease (NAFLD). Our aim was to assess whether pharmacological attenuation of macrophages can be achieved by imatinib, an anti-leukemia drug with known anti-inflammatory and anti-diabetic properties, and how this impacts on NAFLD. We analyzed the pro- and anti-inflammatory gene expression of murine macrophages and human monocytes *in vitro* in the presence or absence of imatinib. In a time-resolved study, we characterized metabolic disease manifestations such as hepatic steatosis, systemic and adipose tissue inflammation as well as lipid and glucose metabolism in obese mice at one and three months of imatinib treatment. Our results showed that imatinib lowered pro-inflammatory markers in murine macrophages and human monocytes *in vitro*. In obese mice, imatinib reduced TNFα-gene expression in peritoneal and liver macrophages and systemic lipid levels at one month. This was followed by decreased hepatic steatosis, systemic and adipose tissue inflammation and increased insulin sensitivity after three months. As the transcription factor sterol regulatory element-binding protein (SREBP) links lipid metabolism to the innate immune response, we assessed the gene expression of SREBPs and their target genes, which was indeed downregulated in the liver and partially in peritoneal macrophages. In conclusion, targeting both inflammatory and lipogenic pathways in macrophages and liver as shown by imatinib could represent an attractive novel therapeutic strategy for patients with NAFLD.

## Introduction

As a result of the increasing prevalence of obesity, non-alcoholic fatty liver disease (NAFLD) has become one of the most common chronic liver diseases worldwide^1^. NAFLD comprises a wide spectrum of diseases ranging from simple fatty liver (NAFL) to non-alcoholic steatohepatitis (NASH), which is characterized by infiltration of immune cells in the liver. In recent years, evidence has accumulated that macrophages play a key role in the onset and progression of NAFLD: Liver injury activates resident liver macrophages leading to cytokine and chemokine release, which induces the recruitment of bone marrow-derived macrophages (BMDM) into the liver, further amplifying the disease process^2–4^. We use the term “liver macrophages” for both bone marrow-derived and resident liver macrophages as their markers strongly overlap^5^. Targeting liver macrophages has been postulated as a therapeutic strategy for NAFLD^3^, especially as currently no specific treatment exists. Pharmacological macrophage depletion indeed prevents the development of NAFLD in mouse models^2,6,7^. Similarly, blocking bone marrow-derived macrophage recruitment to the liver by pharmacological or genetic ablation of different chemokine or cytokine pathways improves NAFLD characteristics (e.g. CCR2-CCL2^4,8–10^, CCR2/5^11,12^, CXCR3-CXCL10^13,14^, CXCL16^15^, IL-6^16^, TNFα^17^). A CCR2/5 antagonist has been tested in a clinical trial with promising results regarding fibrosis in NASH patients^18^. Besides macrophage depletion and blocking macrophage recruitment, pharmacological attenuation of pro-inflammatory macrophages could be an alternative strategy to treat NAFLD. As macrophage activation and chronic low-grade inflammation are linked to metabolic disease and insulin resistance^19^, targeting pathologically activated macrophages might even have a broader impact on metabolic disease manifestations.

A potential candidate drug with anti-inflammatory properties is the tyrosine kinase inhibitor (TKI) imatinib, which was originally developed to target the tumor-associated fusion protein BCR-Abl in chronic myelogenous leukemia (CML). Over the years, several other targets of imatinib have been identified^20^. Most recently, imatinib was shown to inhibit posttranslational phosphorylation of PPARγ^21^, which is an important regulator of macrophage polarization^22^. Anti-inflammatory effects upon imatinib treatment include adoption of an anti-inflammatory phenotype in tumor-associated macrophages^23^, suppressed glycolysis as an indication for anti-inflammatory polarization in leukemia cells^24^, reduced acute liver injury^25^, and attenuated adipose tissue inflammation in obese mice^21^. Additionally, glucose-lowering effects have been observed as “side effects” in cancer patients treated with imatinib^26,27^. In diabetic mouse models, these anti-diabetic effects have been attributed to reduced β-cell death and maintained β-cell function^28–30^. The combination of anti-inflammatory and anti-diabetic effects is reminiscent of PPARγ-agonists/thiazolidinediones (TZDs), which are anti-diabetic drugs also known to dampen macrophage activation^31,32^. TZDs were even implicated in reduced hepatic steatosis via modulation of liver macrophages^33^. However, due to side effects such as fluid retention, congestive heart failure, weight gain and bone fractures, TZDs have been largely abandoned from clinical practice.

Based on the anti-inflammatory and anti-diabetic effects of imatinib potentially involving PPARγ, the aim of our study was to assess whether imatinib directly attenuates macrophages and could therefore be used in disease states with pathological macrophage activation such as NAFLD. We set out a proof-of-concept study to addresses this novel therapeutic concept by testing the effect of imatinib on (1) macrophage activation *in vitro*, (2) NAFLD and other metabolic disease manifestations in a time-resolved manner *in vivo*, and (3) human monocytes to assess its translational application. The concept of pharmacological macrophage attenuation in NAFLD is intriguing as restoring pathologically activated macrophages could potentially not only target the root cause of NAFLD, but also other metabolic disease manifestations such as adipose tissue and systemic inflammation and insulin resistance. A more profound understanding of macrophage modulation and the molecular pathways involved holds the promise for new treatment strategies in NAFLD and metabolic disease.

## Results

### Imatinib lowers pro-inflammatory macrophage activation *in vitro*

**Figure 1:**
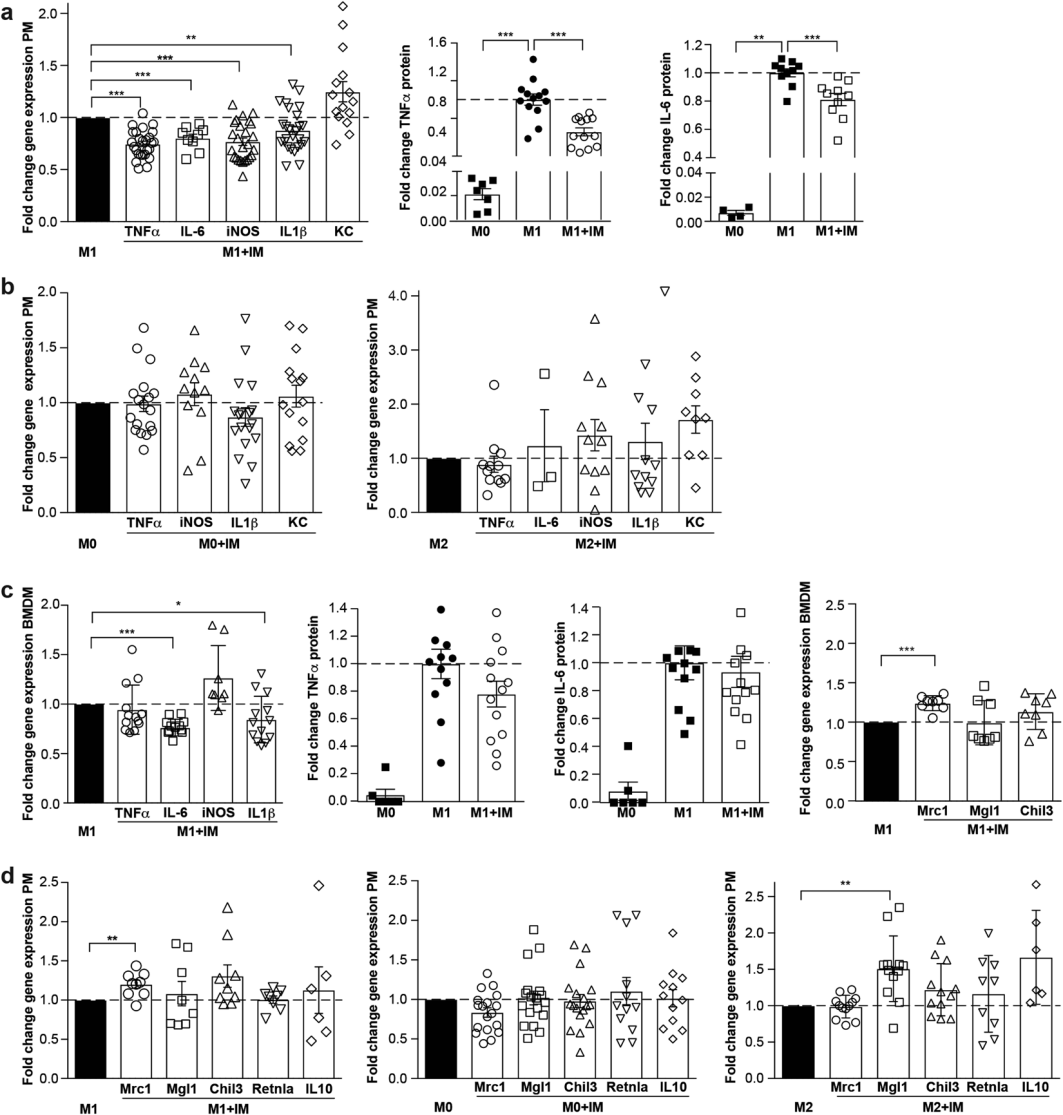
Imatinib lowers pro-inflammatory macrophage activation *in vitro*. (**a**) Fold change gene expression of pro-inflammatory markers in M1-peritoneal macrophages treated with 1 μM of imatinib (M1 + IM, open bars) compared to non-treated M1-controls (M1, closed bar) (n = 8–26). Fold change of TNFα and IL-6 protein in the supernatant of unstimulated (M0), activated (M1) and concomitantly activated/imatinib-treated (M1 + IM) peritoneal macrophages (n = 4–13). (**b**) Fold change of pro-inflammatory gene expression in unstimulated/imatinib-treated (M0 + IM, n = 12–18) and anti-inflammatory/imatinib-treated macrophages (M2 + IM, n = 3–12) compared to their respective controls (M0 or M2). (**c**) Fold change of pro-inflammatory gene expression in activated/imatinib-treated BMDM (M1 + IM) and controls (M1) (n = 8–13). Fold change of TNFα and IL-6 protein in the supernatant of unstimulated (M0), activated (M1) and concomitantly activated/imatinib-treated (M1 + IM) BMDM (n = 6–13). Gene expression of anti-inflammatory genes in BMDM (n = 6–9) treated with imatinib compared to non-treated M1-controls. (**d**) Gene expression of anti-inflammatory genes in M1-activated (n = 6–9), unstimulated M0 (n = 12–18) and anti-inflammatory M2-peritoneal macrophages (n = 6–12) treated with imatinib compared to their respective controls (M1, M0, M2). Data are presented as mean ± SEM. *p < 0.05, **p < 0.01, ***p < 0.001.

Imatinib was tested in differentially activated peritoneal macrophages *in vitro* (M0, M1, M2) after the optimization of housekeeping genes (HKGs), the timing of macrophage activation and the dose of imatinib (Supplementary Fig. S1): In peritoneal M1-activated macrophages, imatinib lowered multiple pro-inflammatory genes, most consistently TNFα. Accordingly, TNFα and IL-6 protein were lower in the supernatants of imatinib-treated M1-macrophages (Fig. 1a). In contrast, pro-inflammatory genes were not altered in unstimulated M0- and anti-inflammatory M2-peritoneal macrophages (Fig. 1b). To confirm this immune-dampening effect in a different macrophage population, imatinib was tested in BMDM, where it exerted a similar immune-dampening effect, although less pronounced than in peritoneal cells (Fig. 1c). To find out whether imatinib only dampens pro-inflammatory genes or also promotes anti-inflammatory gene expression, anti-inflammatory genes were similarly assessed in differentially activated macrophages. We found higher Mgl1 in M2- and Mrc1 in M1-, but no change in M0-macrophages upon imatinib (Fig. 1d). This demonstrates that imatinib primarily lowers pro-inflammatory markers in M1-activated macrophages *in vitro* and does not promote up-regulation of anti-inflammatory genes.

### Imatinib lowers peritoneal macrophage activation in acute inflammation and metabolic disease models

**Figure 2:**
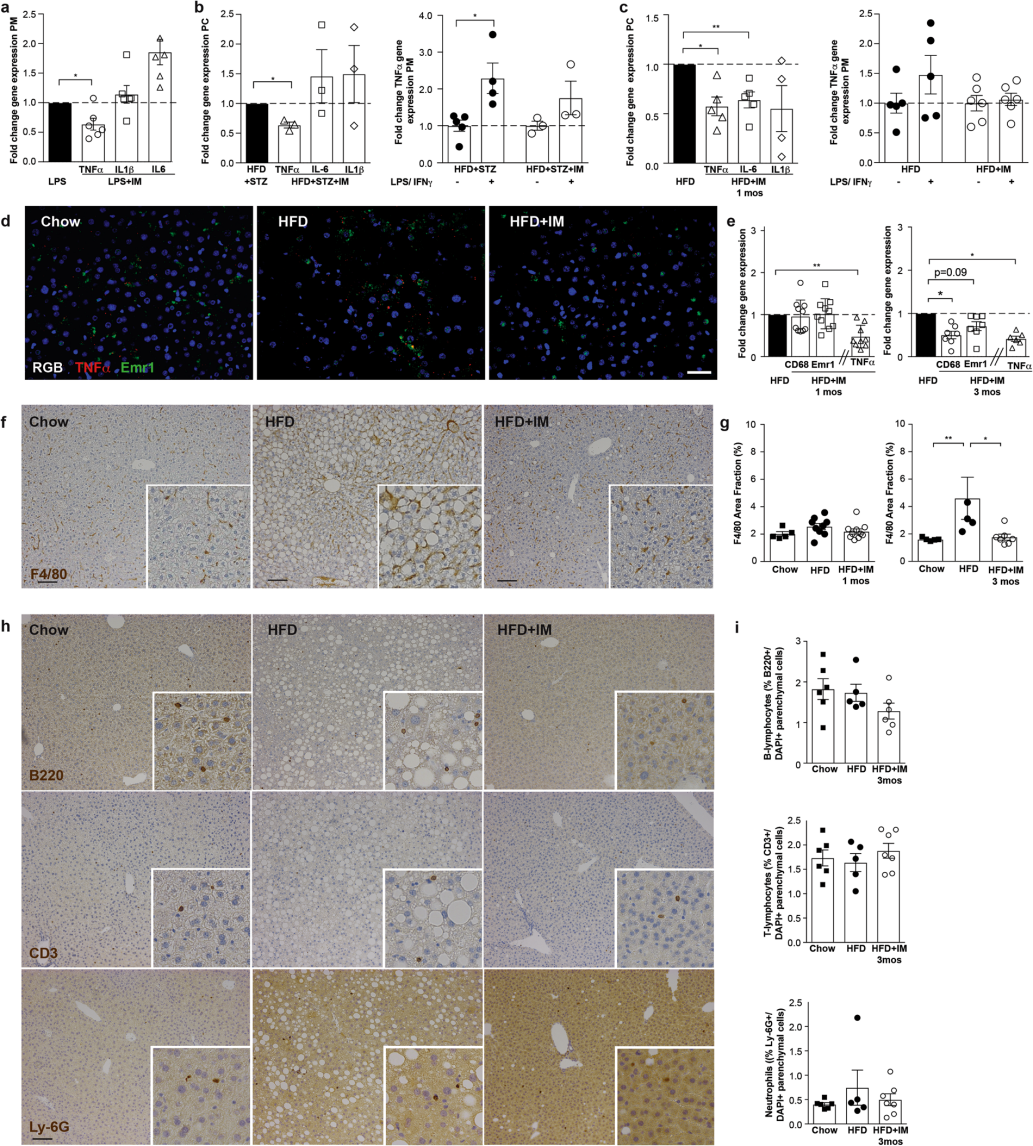
Imatinib lowers pro-inflammatory activation in peritoneal and liver macrophages *in vivo*. (**a**) Fold change of pro-inflammatory genes in peritoneal macrophages in the acute inflammation model (LPS + IM: imatinib pretreated mice; LPS: water treated controls; n = 4–6). (**b**) Fold change of pro-inflammatory genes in peritoneal cells and in peritoneal macrophages upon *ex vivo* stimulation with LPS/IFNγ from diabetic mice treated for one month with imatinib (HFD + STZ + IM) compared to water-treated controls (HFD + STZ) (n = 3–6). (**c**) Fold change of pro-inflammatory genes in peritoneal cells and in peritoneal macrophages upon *ex vivo* LPS/IFNγ-stimulation from obese mice (HFD + IM) treated with imatinib compared with water-treated controls (HFD) (n = 4–10). (**d**) *In situ* hybridization for TNFα (red) and Emr1 (green) mRNA and DAPI nuclear staining (blue) in liver sections from chow, HFD and HFD + IM-treated mice. (**e**) Fold change gene expression of macrophage markers CD68 and Emr1 and TNFα in HFD + IM-treated mice compared to HFD controls after one and three months of imatinib (n = 5–7). (**f,g**) Representative liver sections stained for F4/80 from chow, HFD-fed and HFD + IM-treated mice and quantification by macrophage area fraction (%) at one and three months of imatinib treatment. (**h,i**) Representative liver sections stained for B220, CD3, Ly-6G and DAPI from chow, HFD-fed and HFD + IM-treated mice and quantification by % of cells/ DAPI + parenchymal cells at three months of imatinib. HFD: High fat diet, IM: imatinib, mos: months, PC: peritoneal cells, PM: peritoneal macrophages. Scale bar represents 100μm. Data expressed as mean ± SEM, *p < 0.05, **p < 0.01.

Next, we asked whether this immune-dampening effect of imatinib on macrophages also occurs *in vivo*. To validate peritoneal macrophages as a direct readout for macrophage attenuation, we tested an acute inflammation model by inducing a highly inflammatory response by intraperitoneal (i.p.) Lipopolysaccharide (LPS-) injection in mice pretreated with imatinib or water. Imatinib pretreatment lowered TNFα gene expression in peritoneal macrophages (Fig. 2a), while anti-inflammatory genes were not altered (Supplementary Fig. S2a). To test imatinib in chronic metabolic disease models, diabetic (high fat diet and streptozocin (HFD + STZ)) and obese mice (HFD) were treated with imatinib (IM) or vehicle for up to three months. In diabetic mice, TNFα gene expression was significantly lower in peritoneal cells (0.64 ± 0.05 fold) and less induced by additional LPS/IFNγ-stimulation when compared to untreated controls (1.8 ± 0.5 and 2.3 ± 0.4 fold, respectively, Fig. 2b). Likewise, TNFα gene expression was lower in peritoneal cells of obese mice after one and three months of imatinib treatment (both time points 0.58 ± 0.1 fold) and less induced in peritoneal macrophages upon additional LPS/IFNγ-stimulation (1.2 ± 0.1 and 2.0 ± 0.3 fold, Fig. 2c). Similar to the acute inflammation model, anti-inflammatory genes were not altered (Supplementary Fig. S2b,c). As an additional readout for macrophage activation, we used Seahorse analysis, which showed lower metabolic oxidation (OCR) in peritoneal macrophages from imatinib-treated mice with significantly lower non-mitochondrial and maximum respiration (Supplementary Fig. S2d), while only minor effects were found for glycolysis. Thus, imatinib lowers pro-inflammatory activation of peritoneal macrophages in acute inflammation and metabolic disease models *in vivo* as assessed by gene expression and metabolic flux.

### Imatinib reduces liver macrophages via modulation of the TNFα-pathway

To assess whether imatinib also affects liver macrophages, which are key drivers of NAFLD, we performed a time-resolved study with HFD-induced obese mice (data summarized in Table 1): Concurrent with TNFα-reduction in peritoneal macrophages, imatinib lowered TNFα gene expression in liver tissue after one month. This was followed by a reduction in macrophage gene expression after three months of imatinib treatment (Fig. 2e). Co-localization of TNFα and Emr1 (macrophage marker) mRNA in the liver indicated that TNFα-reduction occurred in liver macrophages (Fig. 2d). Consistent with gene expression, F4/80 area fraction showed no change after one month, but prevention of the HFD-induced increase in liver macrophages after three months of imatinib treatment (Fig. 2f,g). The immune-modulation was specific to macrophages, as other immune cells such as B- and T-lymphocytes and neutrophils were not affected in the liver (Fig. 2h,i). Thus, imatinib leads to early TNFα-reduction in liver macrophages, which later prevents the HFD-induced increase in liver macrophages.

**Table 1:** Summary time resolved study (1 and 3 months imatinib) in obese mice.

	1 month IM	3 months IM
**Peritoneal cells**		
**TNFα gene expression**	**0.58 ± 0.1**	0.58 ± 0.1
**SREBP1c target genes**	**1/4 reduced**	0/4 reduced
pS273 PPARγ-related genes	0/12 induced	1/12 induced
**Liver**		
**TNFα gene expression**	**0.48 ± 0.1**	**0.41 ± 0.1**
**SREBP1a target genes**	**1/3 reduced**	
**SREBP1c target genes**	**3/8 reduced**	1/8 reduced
**SREBP2 target genes**	**2/6 reduced**	1/6 reduced
CD68 gene expression	0.95 ± 0.1	**0.50 ± 0.1**
F4/80 gene expression	1.0 ± 0.1	0.71 ± 0.1 (trend)
F4/80 area fraction	0.86 ± 0.1	**0.38 ± 0.1**
NAS-score	0.9 ± 0.2	**0.42 ± 0.2**
Alkaline phosphatase	not detected	**0.59 ± 0.1**
**Systemic**		
**Cholesterol**	**0.75 ± 0.05**	**0.7 ± 0.1**
**HDL**	**0.77 ± 0.05**	**0.76 ± 0.1**
Triglycerides	1.05 ± 0.1	**0.8 ± 0.1**
TNFα	0.9 ± 0.05	**0.43 ± 0.1**
**Adipose tissue**		
TNFα gene expression	0.55 ± 0.2	**0.39 ± 0.1**
CD68 gene expression	0.93 ± 0.2	0.57 ± 0.2
F4/80 gene expression	0.88 ± 0.1	**0.48 ± 0.1**
pS273 PPARγ-related genes	0/17 induced	5/17 induced
**Metabolic tests**		
ITT	ns	**s**
GTT	ns	ns

Statistical differences between one and three months data are indicated in bold font. Data presented as mean ± SEM.

### Early TNFα-reduction in macrophages is accompanied by changes in lipid metabolism, followed later by markedly decreased hepatic steatosis

**Figure 3:**
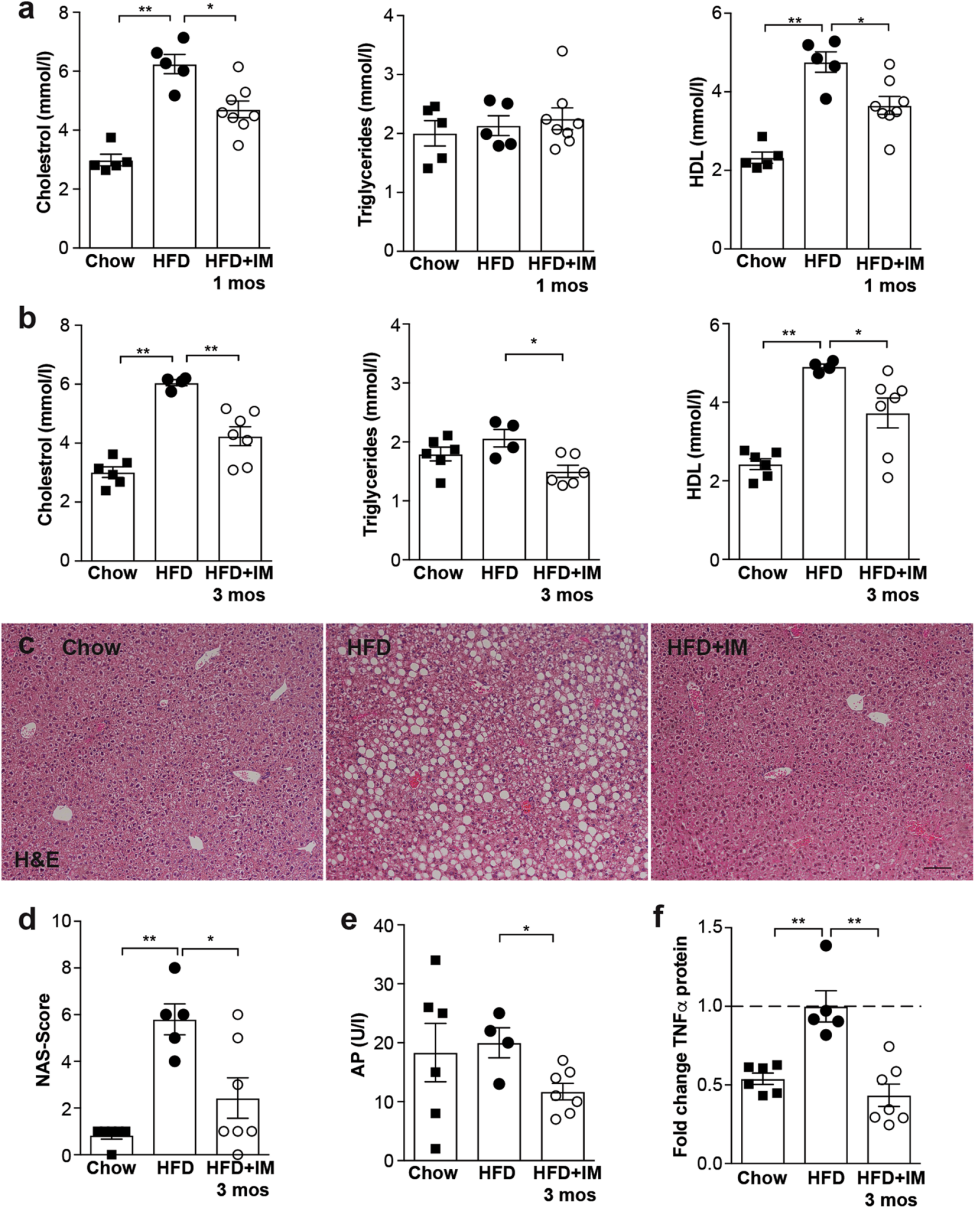
Early TNFα-reduction in macrophages is accompanied by changes in lipid metabolism, followed later by markedly decreased hepatic steatosis. (**a**,**b**) Plasma cholesterol, triglycerides and High-density lipoprotein (HDL) in chow, HFD and HFD + IM-treated mice after one (**a**) and three months of imatinib treatment (**b**; n = 4–7). (**c**) Representative H&E liver stainings from chow, HFD-fed and HFD + IM-treated mice. (**d**) Quantification of NAFLD features by the NAS-score in chow, HFD and HFD + IM-treated mice. (**e**) Alkaline phosphatase (AP) in chow, HFD and HFD + IM-treated mice (n = 4–7). (**f**) Fold change of systemic TNFα protein in chow, HFD and HFD + IM-treated mice (n = 5–7). HFD: High fat diet, IM: imatinib, mos: months. Scale bar represents 100 μm. Data expressed as mean ± SEM, *p < 0.05, **p < 0.01.

We used our time-resolved approach to address the question whether and in what time frame liver macrophage attenuation by imatinib impacts on liver outcomes: Besides TNFα-reduction in the liver, the earliest change we observed were lowered systemic lipid levels after one month of imatinib treatment, also persisting at three months (Fig. 3a,b). All other tissue changes were only observed after three months of imatinib treatment (Table 1): The stark increase in hepatic steatosis in mice on HFD compared to chow was almost completely resolved after three months of imatinib treatment as histologically quantified by the NAFLD activity score (NAS-)score (Fig. 3c,d; Supplementary Fig. S3). Fibrosis was not induced by our obesity model and therefore did not affect the NAS-score. These morphological changes also affected liver function as shown by lower alkaline phosphatase after three months of imatinib (Fig. 3e). Additionally, three months of imatinib prevented the HFD-induced increase in plasma TNFα-levels (Fig. 3f). Thus, besides TNFα-reduction in the liver imatinib leads to early changes in lipid metabolism, which is later followed by markedly decreased hepatic steatosis.

### Imatinib lowers adipose tissue inflammation and increases insulin sensitivity after three months

**Figure 4:**
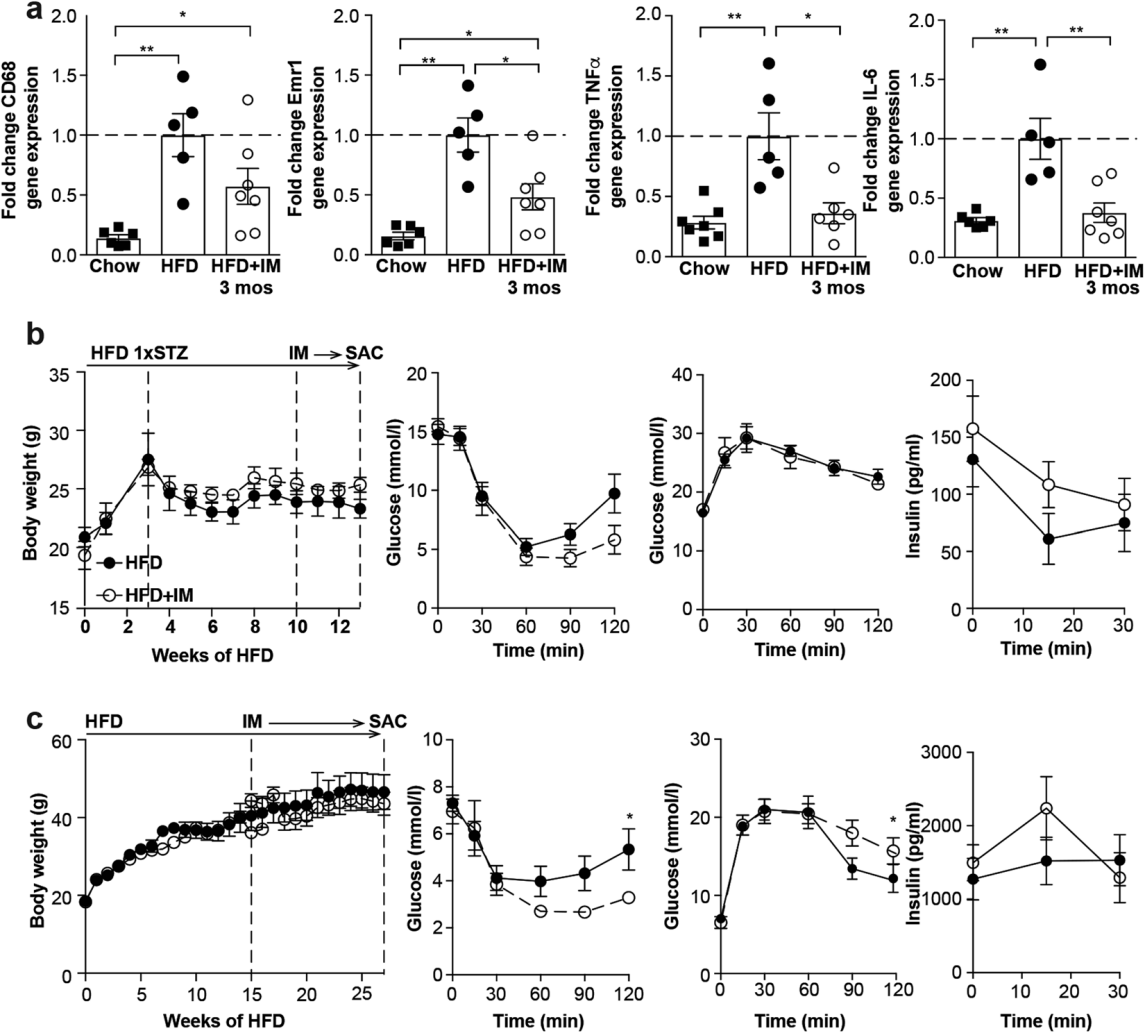
Imatinib reduces adipose tissue inflammation and increases insulin sensitivity over time. (**a**) Fold change gene expression of macrophage markers (CD68, F4/80) and pro-inflammatory M1-markers (TNFα, IL-6) in whole adipose tissue of chow, HFD and HFD + IM-treated animals (n = 5–7). (**b**) Body weight, insulin sensitivity and glucose tolerance with insulin in diabetic mice treated for one month with imatinib (HFD + STZ + IM) and controls (HFD + STZ; n = 4–5). (**c**) Body weight, insulin sensitivity and glucose tolerance with insulin in obese mice treated for three months with imatinib (HFD + IM) and controls (HFD; n = 5–6). HFD: High fat diet, IM: imatinib, mos: months, SAC: sacrifice, STZ: Streptozocin. Data expressed as mean ± SEM, *p < 0.05, **p < 0.01.

We also studied the effect of imatinib on other metabolic disease manifestations such as adipose tissue inflammation and glucose metabolism in a time-resolved manner (Table 1): While there was no change in adipose tissue inflammation after one month of imatinib, the HFD-induced increase in macrophages and pro-inflammatory markers in adipose tissue was reversed by three months of imatinib treatment (Fig. 4a). Flow cytometry confirmed a higher frequency of macrophages in the adipose tissue of HFD-fed animals compared to mice treated for 3 months with imatinib. However, absolute numbers of adipose tissue subpopulations were unchanged (Supplementary Fig. S4). In terms of glucose metabolism, diabetic mice had slightly increased insulin sensitivity after one month of imatinib when compared to vehicle-treated mice, while glucose tolerance was unaltered (Fig. 4b). After one month of treatment, obese mice showed no change in body weight or glucose and insulin tolerance. However, after three months a comparable pattern as in the diabetic model was observed with increased insulin sensitivity, yet unchanged glucose tolerance (Fig. 4c). Taken together, reduced adipose tissue inflammation and increased insulin sensitivity occur after up to three months of imatinib treatment.

### Time-resolved assessment of transcription factors suggests that imatinib targets SREBP, while restoration of PPARγ-phosphorylation is a secondary phenomenon

**Figure 5:**
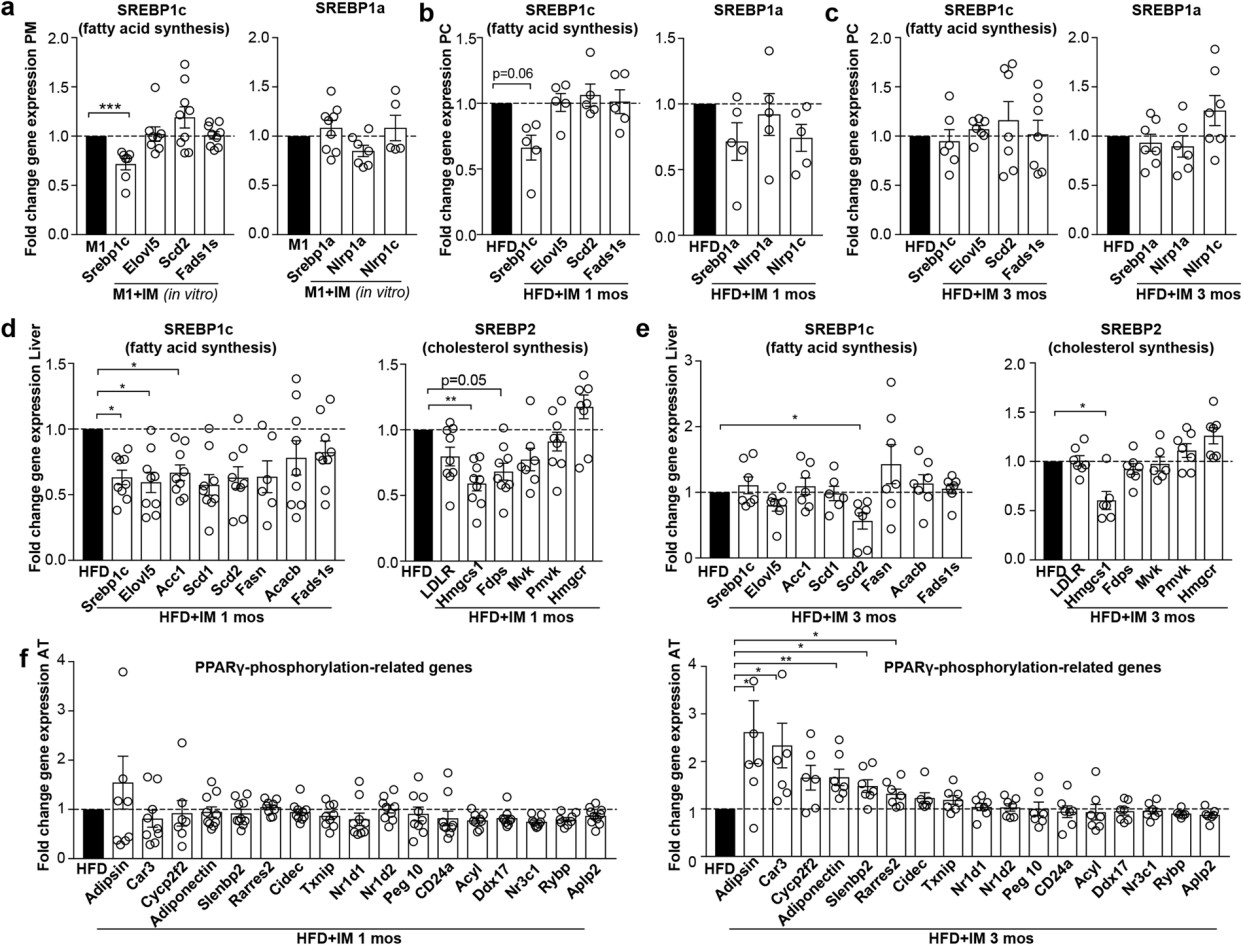
SREBP target genes are downregulated upon imatinib treatment, while PPARγ-phosphorylation seems to be a secondary phenomenon. (**a**) Fold change gene expression of SREBP target genes in M1-stimulated peritoneal macrophages treated with or without imatinib (IM) *in vitro* (n = 9). (**b**,**c**) Fold change gene expression of SREBP target genes in peritoneal cells after one and three months of imatinib treatment (n = 4–7). (**d**,**e**) Fold change gene expression of SREBP target genes in whole liver tissue after one and three months of imatinib (n = 5–9). (**f**) Fold change gene expression of PPARγ-phosphorylation-regulated genes in obese mice treated with imatinib (HFD + IM) compared with water-treated controls (HFD) after one (left) and three months of imatinib treatment (right; n = 5–10). AT: adipose tissue, HFD: High fat diet, IM: imatinib, mos: months, PC: peritoneal cells, PM: peritoneal macrophages. Data expressed as mean ± SEM, *p < 0.05, **p < 0.01, ***p < 0.001.

As the transcription factor sterol regulatory element-binding protein (SREBP) links lipid metabolism to the innate immune response^34^ that were both early affected with imatinib treatment, we assessed the target genes of the three isoforms SREBP1a, SREBP1c and SREBP2: Imatinib downregulated SREBP1c gene expression in cultures of peritoneal macrophages, which was corroborated *in vivo* after one month of imatinib treatment (Fig. 5a,b). SREBP1a and its target genes Nlrp1a and Nlrp1c, in contrast, were not altered in peritoneal cells. In the liver, 3/8 SREBP1c and 2/6 SREBP2 target genes were significantly downregulated after one month imatinib (Fig. 5d). Interestingly, after three months of imatinib treatment, SREBP target gene expression was mostly normalized to baseline in both peritoneal cells and liver tissue (Fig. 5c,e), suggesting that compensatory mechanisms might occur over time.

Another transcription factor that has been shown to control both inflammatory responses and lipid metabolism is PPARγ^35^. In the context of metabolic disease, PPARγ has been shown to become phosphorylated at serine273, leading to dysregulation of a large number of metabolically important genes^36^. Imatinib was shown to inhibit PPARγ-phosphorylation at serine273, thereby reducing insulin resistance and promoting browning of white adipose tissue^21^. We therefore assessed genes related to PPARγ-phosphorylation at serine273 in our time-resolved study. In white adipose tissue, PPARγ-phosphorylation-related genes were dysregulated (lowered) by HFD mainly at the three months’ time point (Supplementary Fig. S5a) when also restoration of these genes occurred by imatinib (upregulation of 5/17 PPARγ-phosphorylation-related genes; Fig. 5f). In peritoneal cells, no changes in PPARγ-phosphorylation-related genes were found at both one and three months of imatinib (Supplementary Fig. S5b), suggesting that phosphorylation at serine273 (pS273) is not directly affected in macrophages. Thus, the early immune-dampening effect in macrophages precedes restored PPARγ-phosphorylation in adipose tissue, indicating that restoration of PPARγ-phosphorylation-related genes might be a secondary phenomenon.

As a last potential mechanism, we assessed genes involved in thermogenesis as imatinib has previously been shown to induce browning of adipose tissue^21^. However, we did not find upregulation of cold-induced thermogenesis genes Pcg1α, Ucp-1, Cox5b, Cpt1b and Dio2 in inguinal adipose tissue of HFD-fed mice upon imatinib treatment (Supplementary Fig. S5c). In sum, our time-resolved assessment of potential pathways suggests that SREBP-signaling is affected in liver and partially in macrophages, while restoration of PPARγ-phosphorylation at pS273 seems to be a secondary phenomenon upon imatinib.

### Imatinib lowers pro-inflammatory activation in human monocytes, but hyperglycemia alters their responsiveness

**Figure 6:**
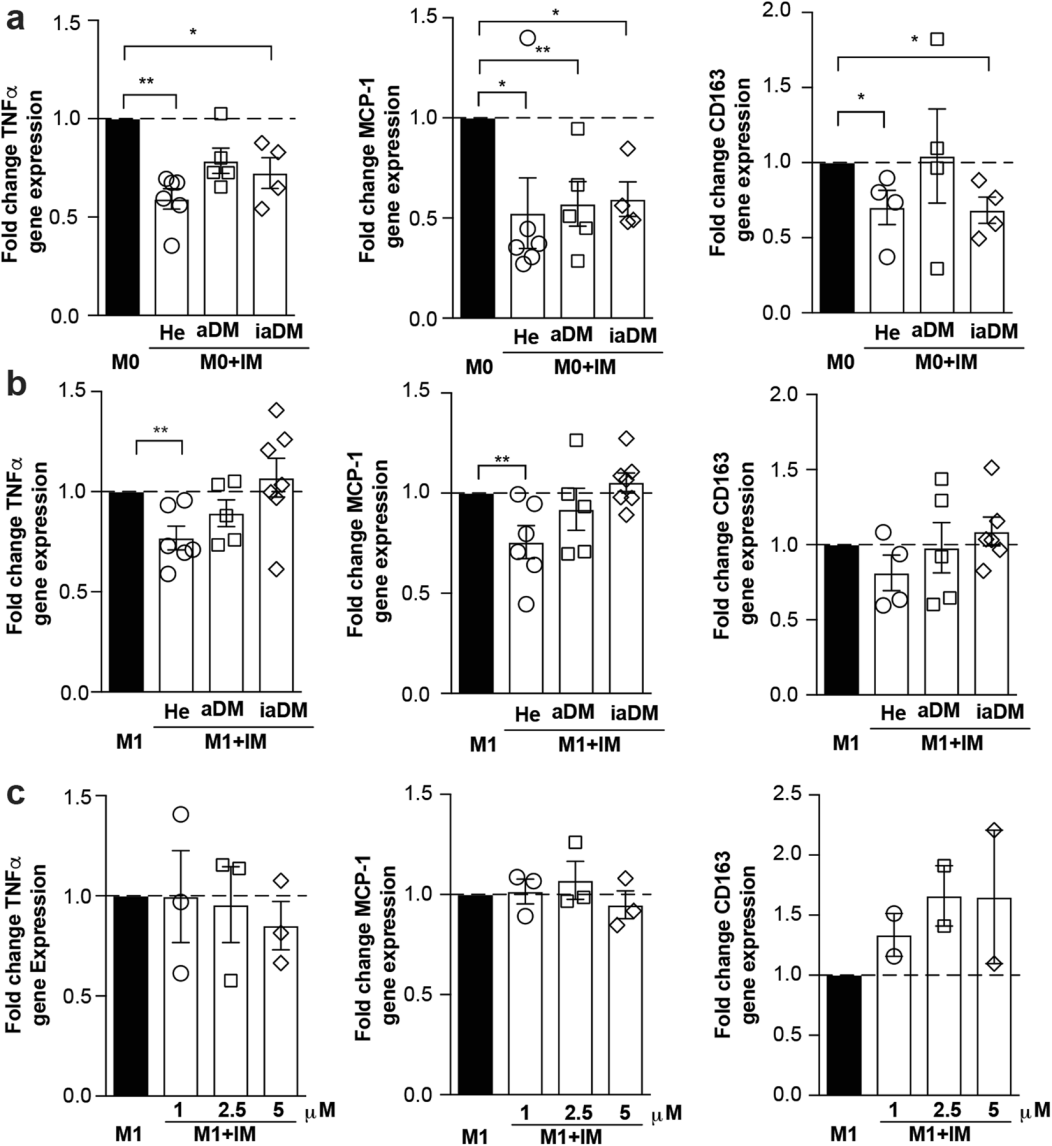
Imatinib lowers pro-inflammatory activation in human monocytes, but hyperglycemia alters their responsiveness. (**a,b**) Fold change gene expression of TNFα, MCP-1 and CD163 in human M0- (**a**) or M1-monocytes (**b**) treated with 1 μM of imatinib (M0 + IM or M1 + IM, open bars) compared to non-treated M0- or M1-monocytes (M0 or M1, closed bar) from healthy controls (He, n = 6), adequately controlled diabetics (aDM, n = 5) and inadequately controlled diabetics (iaDM, n = 5). (**c**) Fold change of gene expression of TNFα, MCP-1 and CD163 with increasing doses of imatinib (1, 2.5, 5 μM) in inadequately controlled diabetics (n = 3). Data expressed as mean ± SEM, *p < 0.05, **p < 0.01.

Finally, we assessed whether immune-modulation by imatinib could also be achieved in human monocytes. As hyperglycemia is known to impact on monocyte activation^37^ and our *in vitro* data showed a differential response to imatinib depending on the activation state, we tested the effect of imatinib on monocytes from subjects with markedly distinct glycemia, including healthy controls (He), diabetics with adequate (aDM) or inadequate glycemic control (iaDM). Supplementary Table S4 shows the baseline characteristics with the intended main differences between aDM and iaDM patients concerning their glycemic control (HbA1c aDM 52.6 ± 5.5 mmol/mol (7.0 ± 0.5%), iaDM 114.5 ± 6.5 mmol/mol (12.6 ± 0.6%)). Imatinib lowered the pro-inflammatory markers TNFα, MCP-1 and CD163 in unstimulated monocytes of all groups, while this immune-dampening effect was gradually lost in M1-activated monocytes with deranged glycemic control (Fig. 6a,b). Increasing the dose of imatinib did not restore the immune-dampening effect in iaDM patients (Fig. 6c). In sum, unstimulated human monocytes respond to imatinib treatment by down-regulation of pro-inflammatory markers. However with deranged glycemic control, the immune-dampening effect of imatinib is lost in activated monocytes, suggesting altered susceptibility to the drug with hyperglycemia.

## Discussion

When first testing the notion of an immune-dampening effect of imatinib on macrophages *in vitro*, we found lower pro-inflammatory gene and protein expression, most consistently TNFα, while anti-inflammatory genes were not upregulated. This was slightly less pronounced in BMDM, most likely due to the artificial differentiation by exogenous Macrophage colony-stimulating factor (M-CSF) over one week. To translate our findings *in vivo*, we performed a time-resolved study assessing the effects of imatinib on macrophages and metabolic disease manifestations in HFD-induced obese mice: Reduction of TNFα in peritoneal and liver macrophages occurred most rapidly upon imatinib. Activated peritoneal macrophages are known to have both enhanced glycolysis and mitochondrial oxidation^38^. Metabolic flux as another measure for macrophage activation confirmed altered polarization by lower metabolic oxidation upon imatinib. In the liver, we were able to localize TNFα in liver macrophages, which decreased over time as shown by lower F4/80 area fraction and CD68 gene expression. Thus, it is conceivable that down-regulation of TNFα by imatinib interrupts the vicious cycle of resident liver macrophage activation and/or bone marrow-derived macrophage recruitment to the liver, subsequently lowering their activation and/or number.

Concomitant with early TNFα-reduction in macrophages, imatinib led to lipid lowering effects, indicative for a mechanism that integrates both innate immunity and lipid metabolism. To assess a common mechanism involving both inflammation and lipogenesis, we first focused on the SREBP transcription factor family, which is known to activate lipogenic transcriptional programs, but has also been shown to control transcriptional regulation that extends beyond lipid synthesis^39^: For example, SREBP1a is highly expressed in immune cells such as macrophages and dendritic cells, where it not only activates genes required for lipogenesis but also a gene encoding Nlrp1, a core component of the inflammasome^34^. Thus, SREBP links lipid metabolism and the innate immune response and could therefore explain the simultaneous effects on inflammation and lipid levels we observed upon imatinib treatment. We found early reductions of SREBP1c-target genes in the liver and partially in peritoneal macrophages. These downregulations were gone after three months of imatinib treatment, most likely due to compensatory mechanisms. We speculate that the SREBP transcriptional program of the target cell determines the phenotypic alteration induced by imatinib: In macrophages, imatinib has preferentially an immune-dampening effect, while in the liver also lipogenesis is affected. Thus, improvements in metabolic disease manifestations likely arise from a combination of the immune-dampening effect on macrophages and lowered lipogenesis induced by imatinib. Hence, in the long-term, lower SREBP target gene expression upon imatinib was associated with reduced hepatic steatosis, systemic and adipose tissue inflammation and increased insulin sensitivity.

Another transcription factor involved in both inflammation and lipid metabolism is PPARγ^35^. In obesity and insulin resistance, PPARγ was shown to become phosphorylated at serine273 with subsequent dysregulation of metabolically important genes^36^. Interestingly, PPARγ-phosphorylation at serine273 was blocked by imatinib, thereby restoring dysregulated diabetes-genes and reducing insulin resistance^21^. However, in our time-resolved assessment the early immune-dampening effect on macrophages and reduction in lipid levels clearly preceded restoration of PPARγ-phosphorylation-related genes in adipose tissue, indicating that restored PPARγ-phosphorylation might be a secondary phenomenon. Restoration of PPARγ-phosphorylation-related genes in adipose tissue potentially develops as less TNFα is available to engage in PPARγ-phosphorylation and its deleterious metabolic downstream effects. A recent study demonstrated that imatinib interferes with the interaction between the histone H3 lysine 4 methyltransferase MLL4 and PPARγ, thereby dampening steatotic target genes in short-term experiments^40^. Thus, dampened SREBP transcriptional programs as observed in our study could ultimately be due to imatinib interfering with the MLL4-PPARγ axis with subsequently reduced transcription of SREBP target genes.

As a third potential mechanistic pathway, we assessed browning of adipose tissue by imatinib, however, we were not able to find consistent upregulation of cold-induced thermogenesis genes. This is in contrast to the publication by Choi and colleagues^21^, which could be explained by differences in the study set-up (administration of imatinib i.p. versus oral; treatment duration and genetic background (C57BL/6 J versus C57BL/6 N)). The latter is insofar important as genetic variability is known to affect cold-induced thermogenesis^41^.

To translate our findings to human disease, we probed the effect of imatinib on human monocytes. Especially, as previous studies showed a heightened inflammatory state in human myeloid cells with hyperglycemia^37,42^, diabetic patients could exhibit altered susceptibility to immune-modulatory drugs like imatinib. Human monocytes responded to imatinib treatment by down-regulation of pro-inflammatory markers also in a non-activated state. This is consistent with previous studies showing that monocytes have a “pre-activated” basal condition that requires only a single stimulation, while macrophages depend on a second signal to be activated^43^. In activated monocytes, however, the immune-dampening effect of imatinib was lost with deranged glycemic control, suggesting altered susceptibility to the drug with hyperglycemia, and could not be overcome with increasing doses of imatinib. Although the extreme stimulation as achieved by *ex vivo* LPS/IFNγ-stimulation might not represent the *in vivo* situation, it uncovers altered susceptibility to immune-modulation with hyperglycemia.

The strength of our study is that the long-term follow-up and time-resolved approach allowed us to distinguish early from later effects of imatinib on different cells and organs. Hence, it became clear that imatinib simultaneously affects inflammatory and lipogenic signals in macrophages and in the liver before reducing metabolic disease manifestations such as NAFLD, systemic and adipose tissue inflammation or insulin resistance. Our findings expand on previous literature by linking SREBP-signaling not only to lipogenesis, but also to innate immunity in the context of NAFLD. A more profound understanding of integrated pathways between inflammation and lipid metabolism could pave the way for the development of novel therapeutics in NAFLD.

The clinical significance of our findings lies in the scarcity of therapeutic measures available for NAFLD patients. Imatinib has generally a mild adverse effect profile and long-term safety record. In rare instances, however, imatinib has been associated with acute liver injury often in connection with hepatotoxic agents interfering with cytochrome P450 enzymes, leading to increased imatinib concentrations^44^. Thus, taking this into account, clinical trials could be envisaged in the context of NAFLD. Imatinib has already been tested in the setting of type 1 diabetes mellitus, however, the results have not yet been published^45^. In the light of our findings that imatinib exerts effects both on innate immunity and lipid metabolism, clinical studies involving patients with metabolic disease – preferentially with chronic low-grade inflammation and NAFLD – could yield promising results in the future.

## Materials and Methods

### Animals

Male C57BL/6 N mice (Charles River Laboratories, Sulzfeld, Germany) were maintained in our SPF-facility at 22 °C room temperature with 12 h light/12 h dark cycle and were housed in groups of 3–5 mice. Body weights were monitored once weekly. Mice used for metabolic experiments were kept for 1 week of acclimation period upon arrival. All procedures were approved by the local Animal Care and Use Committee (Veterinary Office Basel, Switzerland) and carried out in accordance with relevant guidelines and regulations.

### Murine macrophages

Peritoneal cells were harvested from 6–8-week old male C57BL/6 N mice by intra-abdominal lavage, cultured overnight and enriched for macrophages by washing away non-adherent peritoneal cells. For BMDM, bone marrow cells were isolated from murine femur and tibia and differentiated by M-CSF (10 ng/mL, PeproTech, London, UK) for 7–9 days. Peritoneal macrophages or BMDM were polarized to a pro- (M1; 10 ng/mL IFNγ, PeproTech, 100 ng/mL LPS *E. coli* 0111:B4, Sigma-Aldrich, Saint Louis, MO, USA) or anti-inflammatory phenotype (M2; 10 ng/mL IL-4 and IL-13, Thermo Fisher Scientific, Waltham, MA, USA) or left unstimulated (M0) in the presence or absence of imatinib (1 μM, Novartis, Basel, Switzerland) for 6 hours.

### Gene expression analysis

RNA was isolated using NucleoSpin RNA kit (Macherey Nagel, Düren, Germany) and RNeasy Plus Universal Mini kit (QIAGEN, Düsseldorf, Germany). Reverse transcription was performed with SuperScriptII Reverse Transcriptase kit (Thermo Fisher Scientific). GoTaq qPCR Master Mix (Promega, Madison, WI, USA) was used for real-time PCR (ViiA7, Thermo Fisher Scientific). Primer sequences (Microsynth, Balgach, Switzerland) are listed in Supplementary Table S1.

### Protein expression analysis

Plasma insulin, TNF-α and IL-6 were quantified by electrochemiluminescence (MESO SECTOR S 600) using kits from MesoScale Diagnostics (MSD, Rockville, MD, USA).

### Acute *in vivo* inflammation model

Imatinib (100 mg/kg) or water was administered by gavage three times during 24 h prior to a single i.p. LPS-injection (1 mg/kg). Analysis was performed 2 h post LPS and peritoneal cells and macrophages assessed by PCR.

### Chronic *in vivo* inflammation model

Mice on high fat diet (HFD containing 58% fat, 16.4% protein and 25.6% carbohydrate, Research diet, New Brunswick, NJ, USA; start at 4–5 weeks of age for 14–27 weeks) and mice on HFD with a single i.p. injection of streptozocin (at week three of HFD STZ 130 mg/kg, Sigma-Aldrich) were treated with oral imatinib (gavage 100 mg/kg) or water for one or three months. A dose of 100 mg/kg imatinib has been reported to reach slightly lower steady state plasma concentrations (1 μM at 8 hours^46^) compared to humans treated with 400 mg imatinib daily (1.46 μM at 24 hours^47^) due to faster clearance in mice. Insulin and glucose tolerance tests (ITT/ GTT) were performed at 4 or 8 weeks of imatinib treatment with blood samplings from the tail vein before and 15, 30, 60 and 90 minutes after i.p. injection of 2 g/kg body weight glucose or 2U/kg body weight insulin. Peritoneal cells and macrophages were harvested as described for *in vitro* experiments. Other readout measures are specified below.

### Seahorse XF flux analysis

Glycolysis (ECAR; extracellular acidification rate) and mitochondrial respiration (OCR, oxygen consumption rate) were measured by XF96 Seahorse Metabolic Analyzer (Seahorse Bioscience, North Billerica, MA, USA) in peritoneal macrophages *ex vivo* as previously described^48^.

### Flow cytometry of adipose tissue macrophages

Epididymal adipose tissue was minced and digested using collagenase IV (Worthington, OH, USA) and DNAse I (Sigma-Aldrich) at 37 °C for 25–30 min. To identify adipose tissue macrophages (ATMs), cells were stained with specific surface markers (Supplementary Table S2) and analyzed using the BD LSRII instrument (BD Biosciences, Franklin Lakes, NJ, USA) and FlowJo software (TreeStar Inc., Ashland, OR, USA). Among single (Singlets), live (DAPI^−^) leukocytes (CD45^+^) and after excluding eosinophils (CD45^+^F4/80^low^SiglecF^+^) ATMs (non-eosinophils CD11b^+^F4/80^+^) were classified as double negative (DN), monocyte-derived M1a (CD11c^+^CD206^−^), inflammatory M1b (CD11c^+^CD206^mid^) and anti-inflammatory M2 (CD11c^− to low^CD206^high^) (gating strategy: Supplementary Fig. S4a–c).

### Liver histology

Hematoxylin-eosin (H&E) was performed according to established protocols and the NAS-score^49^ assessed in a blinded fashion. Immunohistochemistry (IHC) for F4/80, CD3, B220, and Ly-6G (antibodies in Supplementary Table S3) was performed on paraffin-embedded liver sections. For quantification of immune cells, liver sections were scanned by a Prior robot/Nikon slide scanner. Three independent visual fields were semi-automatically quantified for area fraction (F4/80) or number of cells per DAPI-positive parenchymal cells (CD3, B220, Ly-6G) using the Nikon software (NIS) tool.

### ***In situ*** hybridization (ISH)

Mouse TNFα (VB1-10175-VT) and Emr1 (VB6-12917-VT) genes were detected in formalin fixed, paraffin embedded (FFPE), 5μm liver sections using the ViewRNA ISH system (Affymetrix, Santa Clara, CA, USA) as previously described^50^. Brightfield and fluorescent images were acquired using a laser scanning confocal microscope (LSM710, Zeiss, Oberkochen, Germany) and Zen2 software (Zeiss) and subjected to image processing with ImageJ software.

### Liver enzymes and lipids

Liver enzymes and blood lipids were measured in mouse plasma using a Cobas 8000 modular analyzer (Roche Diagnostics, Basel, Switzerland) according to the manufacturer’s protocol.

### Human monocytes

Study approval was obtained from the local ethics committee (Ethics Committee of northwest and central Switzerland, EKNZ). The human study was conducted in accordance with the Declaration of Helsinki and relevant guidelines and regulations. All diabetic subjects (HbA1c > 6.5%) and healthy volunteers (BMI 18–25 kg/m^2^) gave written, informed consent. Detailed medical history and baseline characteristics were obtained at the day of the blood draw. Monocytes were enriched using MagniSort® Human Pan-Monocyte Enrichment kit (Thermo Fisher Scientific) from peripheral blood mononuclear cells (PBMCs). Human monocytes were kept for 2 h to attach and then activated towards a pro-inflammatory phenotype (M1; 10 ng/mL IFNγ, ImmunoTools, Friesoythe, Germany, 100 ng/mL Lipopolysaccharide (LPS) *E. coli* 0111:B4, Sigma-Aldrich, Saint Louis, MO, USA) or left unstimulated (M0) in the presence or absence of imatinib (1 μM) for 24 h.

### Data analysis

Data are expressed as mean ± SEM. Unpaired Mann-Whitney test was used for statistical significance (GraphPad Prism). A p-value < 0.05 was considered as statistically significant.

## Electronic supplementary material

Supplementary information
