# Supplementary material for: Imatinib reduces non-alcoholic fatty liver disease in obese mice by targeting inflammatory and lipogenic pathways in macrophages and liver
Source: Sci Rep. 2018 Oct 17;8:15331. doi: 10.1038/s41598-018-32853-w (PMC6193017; doi:10.1038/s41598-018-32853-w)
Supplement: Supplementary file 1 — Supplementary information [file 41598_2018_32853_MOESM1_ESM.docx]

**August 5^th^, 2018**

**Imatinib reduces non-alcoholic fatty liver disease in obese mice**

**by targeting inflammatory and lipogenic pathways in macrophages and liver**

Imatinib in non-alcoholic liver disease

**Shefaa AlAsfoor**^1^**, Theresa V. Rohm**^1^**, Angela J. T. Bosch**^1^**, Thomas Dervos**^1^**, Diego Calabrese**^2^**, Matthias S. Matter**^3^**, Achim Weber**^4^**, Claudia Cavelti-Weder**^1^

^1^Clinic of Endocrinology, Diabetes and Metabolism, University Hospital Basel, Basel, Switzerland, and Department of Biomedicine, University of Basel, Basel, Switzerland

^2^Department of Biomedicine, University of Basel, Basel, Switzerland

^3^Institute of Pathology, University Hospital of Basel, Basel, Switzerland

^4^Department of Pathology and Molecular Pathology, University and University Hospital of Zurich, Zurich, Switzerland

**Corresponding author**:

Claudia Cavelti-Weder MD MPH

University Hospital of Basel, Department of Biomedicine

Hebelstrasse 20, 4031 Basel, Switzerland

Phone: +41 61 328 63 23, Fax: +41 61 265 51 00

[claudia.cavelti-weder@usb.ch](mailto:claudia.cavelti-weder@usb.ch)

**SUPPLEMENTARY TABLES**

| **Gene** | **Forward Primer** | **Reverse Primer** |
| --- | --- | --- |
| **Mouse HKG and M1- and M2 -markers** | | |
| **B2m** | 5′ TTCTGGTGCTTGTCTCACTGA | 5′ CAGTATGTTCGGCTTCCCATTC |
| **Ppia** | 5′ GAGCTGTTTGCAGACAAAGTTC | 5′ CCCTGGCACATGAATCCTGG |
| **TNF-α** | 5′ ACTGAACTTCGGGGTGATCG | 5′ TGAGGGTCTGGGCCATAGAA |
| **IL-6** | 5′ GGATACCACTCCCAACAGACCT | 5′ GCCATTGCACAACTCTTTTCTC |
| **IL-1β** | 5′ GCAACTGTTCCTGAACTCAACT | 5′ ATCTTTTGGGGTCCGTCAACT |
| **iNOS** | 5′ GTTCTCAGCCCAACAATACAAGA | 5′ GTGGACGGGTCGATGTCAC |
| **KC** | 5′ CTGGGATTCACCTCAAGAACATC | 5′ CAGGGTCAAGGCAAGCCTC |
| **Mrc1** | 5′ CTCTGTTCAGCTATTGGACGC | 5′ CGGAATTTCTGGGATTCAGCTTC |
| **Mgl1** | 5′ TGAGAAAGGCTTTAAGAACTGGG | 5′ GACCACCTGTAGTGATGTGGG |
| **Rentla** | 5′ CCAATCCAGCTAACTATCCCTCC | 5′ CCAGTCAACGAGTAAGCACAG |
| **Chil3** | 5′ AGGAAGCCCTCCTAAGGACA | 5′ CTCCACAGATTCTTCCTCAAAAGC |
| **IL-10** | 5′ AGGCGCTGTCATCGATTTCTC | 5′ GCCTTGTAGACACCTTGGTCTT |
| **CD68** | 5′ GCAGCACAGTGGACATTCAT | 5′ AGAGAAACATGGCCC GAAGT |
| **Adgre1**  **(F4/80 or Emr1)** | 5′ GCC CAG GAGTGGAATGTCAA | 5′ CAGACACTCATCAACATCTGCG |
| **Mouse sterol regulatory element-binding protein (SREBPs) genes** | | |
| **Srebp1a** | 5′ GCCGGCGCCATGGACGAGCTGGCC | 5′ CAGGAAGGCTTCCAGAGAGGAGGC |
| **Nlrp1a** | 5′ AGGCTCTTTACCCTCTTCTA | 5′ ATGTGCTTCTTCTTCTGGTA |
| **Nlrp1c** | 5′ GAATCTTTACTCCACCCAGC | 5′ CTTTTCCTGGCAAATGTCTT |
| **Srebp1c** | 5′ GGAGCCATGGATTGCACATT | 5′ GGCCCGGGAAGTCACTGT |
| **Elovl5** | 5′ CTGAGTGACGCATCGAAATG | 5′ CTTGCACATCCTCCTGCTC |
| **Scd2** | 5′ TGCCTTGTATGTTCTGTGGC | 5′ TCCTGCAAGCTCTACACCTG |
| **Fads1s** | 5′ TGGTGCCCTTCATCCTCTGT | 5′ GGTGCCCAAAGTCATGCTGTA |
| **Acc1** | 5′ CCTCCGTCAGCTCAGATACA | 5′ TTTACTAGGTGCAAGCCAGACA |
| **Scd1** | 5′ CTGTACGGGATCATACTGGTTC | 5′ GCCGTGCCTTGTAAGTTCTG |
| **Fasn** | 5′ AGCGGCCATTTCCATTGCCC | 5′ CCATGCCCAGAGGGTGGTTG |
| **Acacb** | 5′ CCCAGGAGGCTGCATTGA | 5′ AGACATGCTGGGCCTCATAGTA |
| **LDLR** | 5′ ACCTGCCGACCTGATGAATTC | 5′ GCAGTCATGTTCACGGTCACA |
| **Hmgcs1** | 5′ TTTGATGCAGCTGTTTGAGG | 5′ CCACCTGTAGGTCTGGCATT |
| **Fdps** | 5′ GAGTCTGCCCGATCTCTGTC | 5′ TGAACCTGCTGGAGCTCTTT |
| **mvk** | 5′ GAAGACATCGTCCCTTGCTG | 5′ AAC CCT TCT GGT GTGGACA |
| **Pmvk** | 5′ GCTCGCATCCAGAAGTCTCT | 5′ GCTCTCTGGTCCACTCAAGG |
| **Hmgcr** | 5′ GGCCTCCATTGAGATCCG | 5′ CACAATAACTTCCCAGGGGT |
| **Mouse PPAR-γ phosphorylation-related genes** | | |
| **Rarres2** | 5′ GCCTGGCCTGCATTAAAATGG | 5′ CTTGCTTCAGAATTGGGCAGT |
| **Txnip** | 5′ TCTTTTGAGGTGGTCTTCAACG | 5′ GCTTTGACTCGGGTAACTTCACA |
| **Nr1d1** | 5′ TACATTGGCTCTAGTGGCTCC | 5′ CAGTAGGTGATGGTGGGAAGTA |
| **CD24a** | 5′ GTTGCACCGTTTCCCGGTAA | 5′ CCCCTCTGGTGGTAGCGTTA |
| **Peg10** | 5′ TGCTTGCACAGAGCTACAGTC | 5′ AGTTTGGGATAGGGGCTGCT |
| **Acyl** | 5′ CAGCCAAGGCAATTTCAGAGC | 5′ CTCGACGTTTGATTAACTGGTCT |
| **Cidec** | 5′ ATGGACTACGCCATGAAGTCT | 5′ CGGTGCTAACACGACAGGG |
| **Nr1d2** | 5′ TGAACGCAGGAGGTGTGATTG | 5′ GAGGACTGGAAGCTATTCTCAGA |
| **Ddx17** | 5′ TCTTCAGCCAACAATCCCAATC | 5′ GGCTCTATCGGTTTCACTACG |
| **Rybp** | 5′ CGACCAGGCCAAAAAGACAAG | 5′ CACATCGCAGATGCTGCATT |
| **Nr3c1** | 5′ AGCTCCCCCTGGTAGAGAC | 5′ GGTGAAGACGCAGAAACCTTG |
| **Aplp2** | 5′ GTGGTGGAAGACCGTGACTAC | 5′ TCGGGGGAACTTTAACATCGT |
| **Slenbp2** | 5′ ATGGCTACAAAATGCACAAAGTG | 5′ CCTGTGTTCCGGTAAATGCAG |
| **Cycp2f2** | 5′ GTCGGTGTTCACGGTGTACC | 5′ AAAGTTCCGCAGGATTTGGAC |
| **Car3** | 5′ TGACAGGTCTATGCTGAGGGG | 5′ CAGCGTATTTTACTCCGTCCAC |
| **Adipsin** | 5′ CATGCTCGGCCCTACATGG | 5′ CACAGAGTCGTCATCCGTCAC |
| **Adiponectin** | 5′ TGTTCCTCTTAATCCTGCCCA | 5′ CCAACCTGCACAAGTTCCCTT |
| **Mouse adipose tissue browning markers** | | |
| **CPT1b** | 5′ TGCCTTTACATCGTCTCCAA | 5′ GGCTCCAGGGTTCAGAAAGT |
| **UCP1** | 5′ CTTTGCCTCACTCAGGATTGG | 5′ ACTGCCACACCTCCAGTCATT |
| **PGC1α** | 5′ TATGGAGTGACATAGAGTGTGCT | 5′ CCACTTCAATCCACCCAGAAAG |
| **Dio2** | 5′ AATTATGCCTCGGAGAAGACCG | 5′ GGCAGTTGCCTAGTGAAAGGT |
| **Cox5b** | 5′ ATCAGCAACAAGAGAATAGTGGG | 5′ GTAATGGGTTCCACAGTTGGG |
| **Human HKG and M1- and M2- markers** | | |
| **B2m** | 5′ GCTCGCGCTACTCTCTCTTT | 5′ TGTCGGATGGATGAAACCCA |
| **Ppia** | 5′ GCATACGGGTCCTGGCATCTTGTCC | 5′ ATGGTGATCTTCTTGCTGGTCTTGC |
| **TNF-α** | 5′ CAGAGGGCCTGTACCTCATC | 5′ GGAAGACCCCTCCCAGATAG |
| **MCP-1** | 5′ CCCCAGTCACCTGCTGTTAT | 5′ TGGAATCCTGAACCCACTTC |
| **Mrc1** | 5′ CGAGGAAGAGGTTCGGTTCACC | 5′ GCAATCCCGGTTCTCATGGC |
| **CD163** | 5′ TTGCCAGCAGCTTAAATGTG | 5′ AGGACAGTGTTTGGGACTGG |

**Supplementary Table 1:** Primers sequences used for quantitative real time-PCR

| **Antibody** | **Clone** | **Fluorophore** | **Source** |
| --- | --- | --- | --- |
| CD45 | 30-F11 | PerCP/Cy5.5 | Biolegend |
| Siglec-F | E50-2440 | BV510 | BD Biosciences |
| CD11b | M1/70 | BV421 | Biolegend |
| F4/80 | BM8 | PE | Biolegend |
| CD11c | N418 | PE/Cy7 | Biolegend |
| CD206 | C068C2 | A647 | Biolegend |

**Supplementary Table 2**: List of Antibodies for flow cytometry in adipose tissue

| **IHC** | **Primary antibody** | **Diluent** | **Visualization** |
| --- | --- | --- | --- |
| **F4/80** | F4/80 T-2006 clone BM8  BMA Biomedicals | 1/50 | Performed on Discovery Ventana UltraMap anti Rat DAB Kit |
| **B220** | B220 553084 clone RA3-6B2,  BD Biosciences | 1/4000 | Performed on Discovery Ventana UltraMap anti Rat DAB Kit |
| **CD3** | CD3 MA1-90582 clone SP7,  Thermo Fisher Scientific | 1/300 | Performed on Bond Leica DAB Kit |
| **Ly-6G** | Ly-6G 551459 clone 1A8,  BD Biosciences | 1/600 | Performed on Bond Leica DAB Kit |

**Supplementary Table 3:** Antibodies for IHC of immune cells in paraffin liver sections

|  | **Healthy Controls**  **(n=6)** | **Diabetics,**  **adequate control**  **(n=5)** | **Diabetics, inadequate control**  **(n=7)** |
| --- | --- | --- | --- |
| **General parameter** |  |  |  |
| Sex (M/F) | 6/0 | 3/2 | 4/3 |
| Age (years) | 29.5±3.3 | 57.0±5.0 | 52.7±5.5 |
| BMI (kg/m^2^) | 22.8±1.5 | 33.5±1.9 | 37.4±4.5 |
| Weight (kg) | 74.8±4.6 | 100.0±7.6 | 111.3±16.8 |
| Waist-to-hip Ratio | 0.86±0.02 | 0.99±0.05 | 1.04±0.05 |
| **Glc metabolism** |  |  |  |
| HbA1c (mmol/mol (%)) | na | **52.6±5.5 (7.0±0.5)** | **114.5±6.5(12.6±0.6)**** |
| Fasting plasma glucose (mmol/l), average | na | **8.3±1.2** | **16.0±1.3**** |
| Diabetes duration (years) | na | 7.2±3.9 | 12.9±4.9 |
| Antidiabetics   - Oral/GLP1-Anal. (%) - Insulin (%) | na  na | 80  20 | 85.7  85.7 |
| **Inflammation** |  |  |  |
| CRP (mg/dl) |  | 3.9±0.6 | 10.0±4.6 |
| Leukocytes (x10^9^/l) |  | **6.4±0.4** | **8.3±0.7*** |
| **Other Cv-Risks** |  |  |  |
| Blood Pressure   - Antihypertensive drug (%) - Systolic (mmHg) - Diastolic (mmHg) | 0  125±4  72±4 | 40  126±9  80±6 | 85.7  143±7  83±5 |
| Family History   - Diabetes (%) - Obesity (%) - CV disease (%) | 16.7  16.7  16.7 | 100  80  40 | 57.1  57.1  42.9 |
| Smoking (%) | 0 | 0 | 42.9 |

**Supplementary Table 4: Baseline characteristics of healthy, adequately and inadequately controlled diabetics.** Statistical differences between adequately and inadequately controlled diabetics are indicated in bold font. Data presented as mean±SEM, na: not applicable, *p<.05, **p<.01.

**SUPPLEMENTARY FIGURES**

**
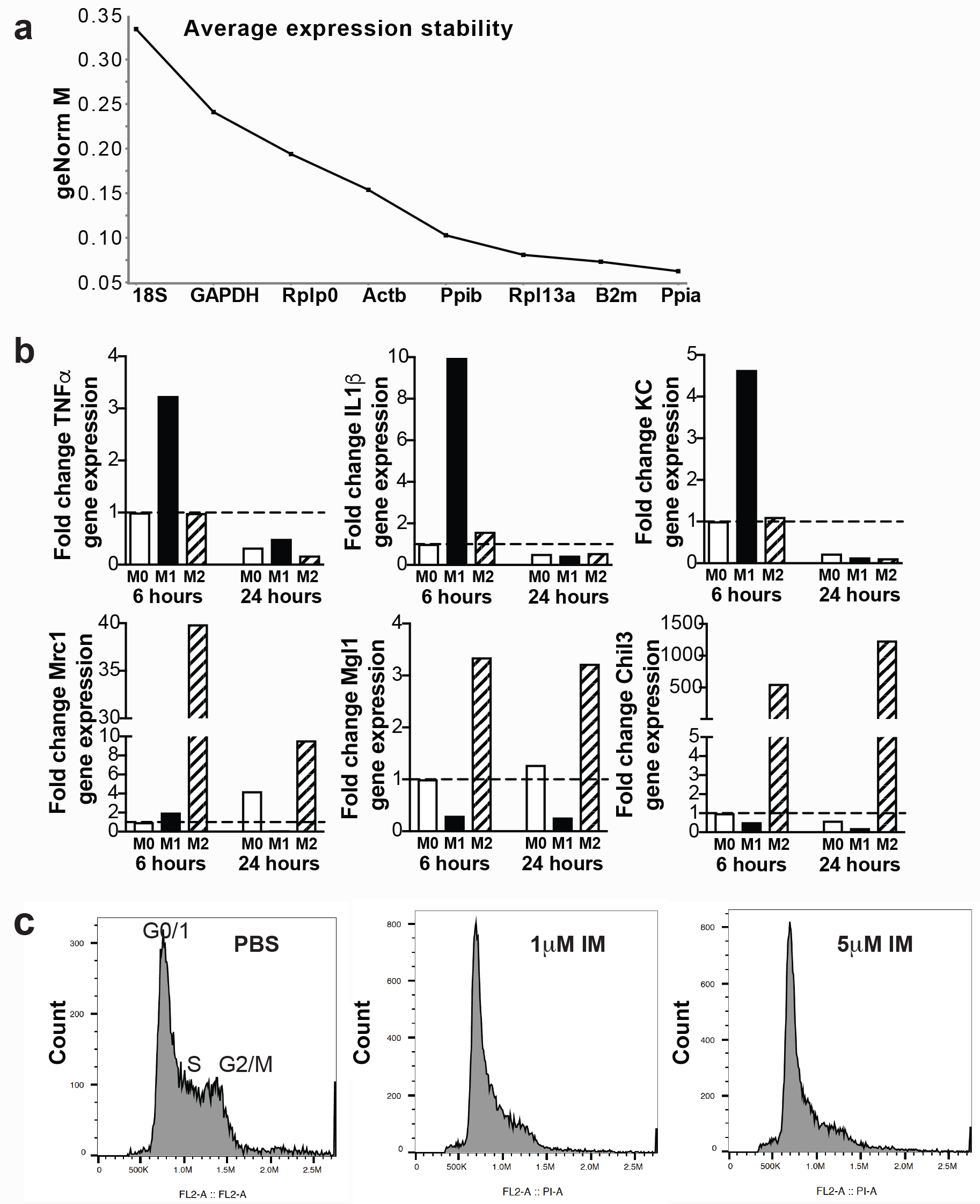
**

**Supplementary Figure 1. Optimization of *in vitro* set-up.** Due to the highly dynamic behavior of macrophages, *in vitro* readouts were optimized for macrophage housekeeping genes (HKGs), the optimal time point for macrophage activation and imatinib dose. (**a**) Average expression stability of HKGs 18S, GAPDH, Rplp0, Actb, Ppib, Rpl13a, B2m, Ppia according to the geNorm algorithm with B2m and Ppia most stably expressed. (**b**) The 6-hour time point was chosen for peritoneal macrophages when fold gene expression of pro-inflammatory M1-markers was most pronounced, while fold gene expression of anti-inflammatory M2-markers was similar at 6 and 24 hours after stimulation. (**c**) Flow cytometry for cell cycle with G1-phase arrest of the CML-cell line K562 at both 1uM and 5uM imatinib compared to PBS-treated cells.

**
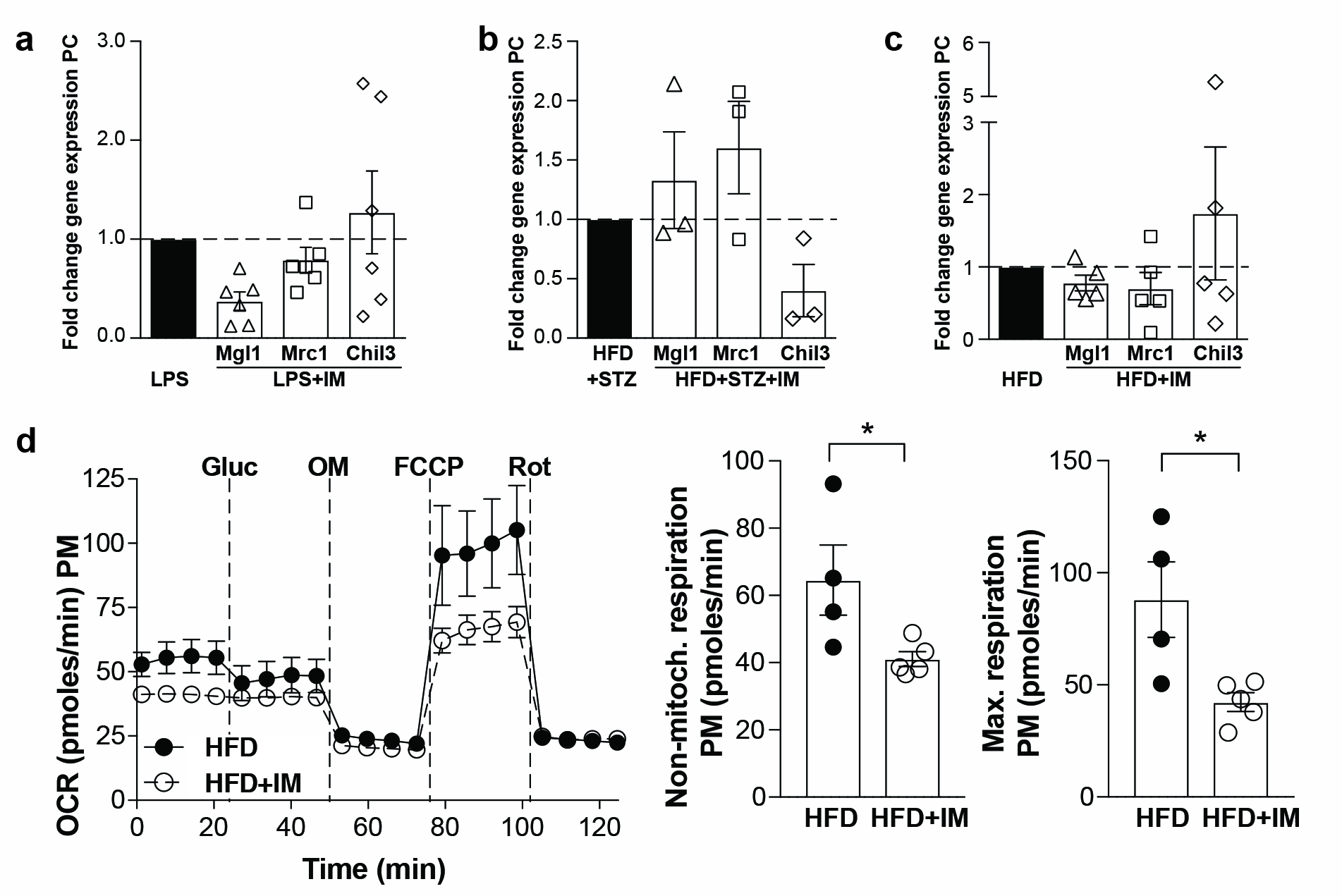
 Supplementary Figure 2. Anti-inflammatory gene expression and metabolic flux in peritoneal macrophages of imatinib-treated mice.** Fold change of anti-inflammatory genes in peritoneal macrophages of the acute inflammation model (**a**), diabetic (**b**) and obese mice (**c**) treated with imatinib compared to their respective controls. (**d**) Seahorse flux analysis with OCR (metabolic oxidation) and calculated non-mitochondrial and maximum respiration (pmoles/min) in peritoneal macrophages of HFD-fed mice treated for 3 months with imatinib or vehicle. Gluc: Glucose, HFD: High fat diet, IM: imatinib, OM: oligomycin, PC: peritoneal cells, PM: peritoneal macrophages, Rot: rotenone, STZ: Streptozocin. Data expressed as mean±SEM, *=p<.05.

**
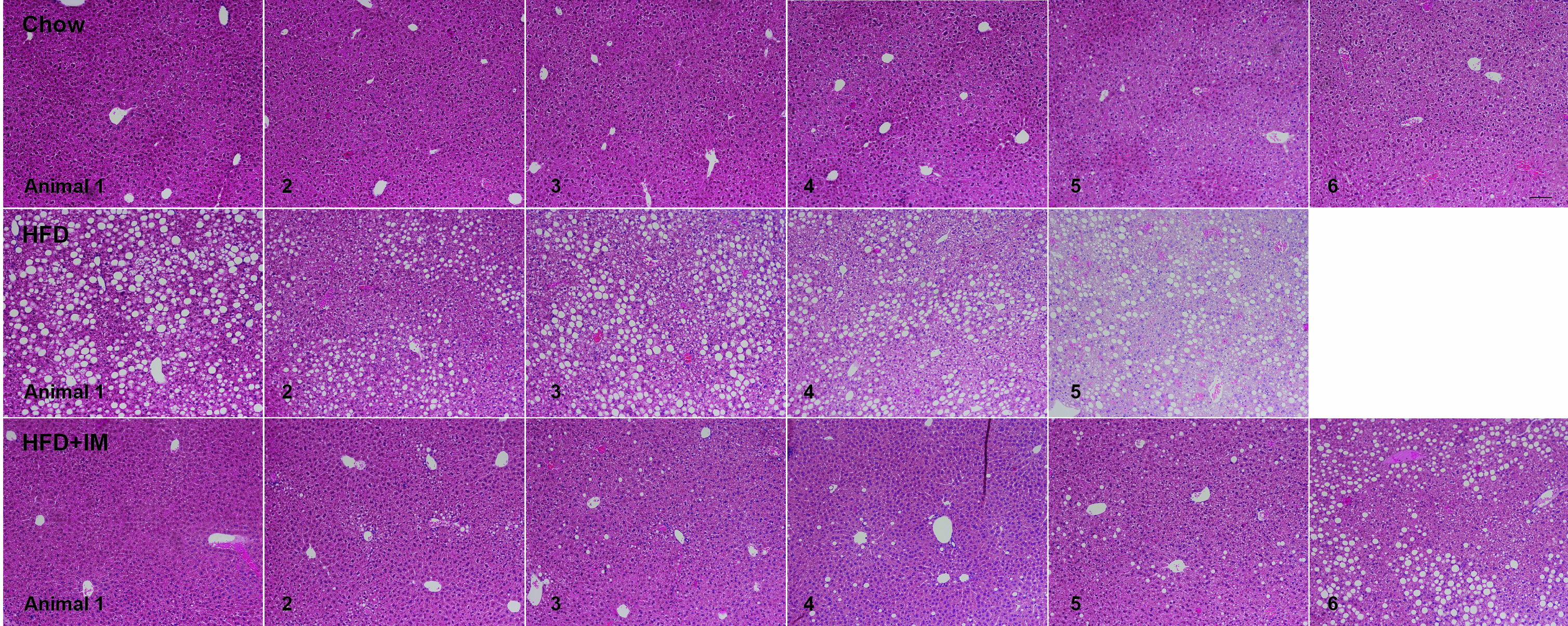
 Supplementary Figure 3. Liver sections of individual mice regarding steatosis.** Representative H&E liver sections of 5-6 individual mice of the chow, HFD and HFD+IM-groups. HFD: high fat diet; IM: imatinib. Scale bar represents 100μm.

**
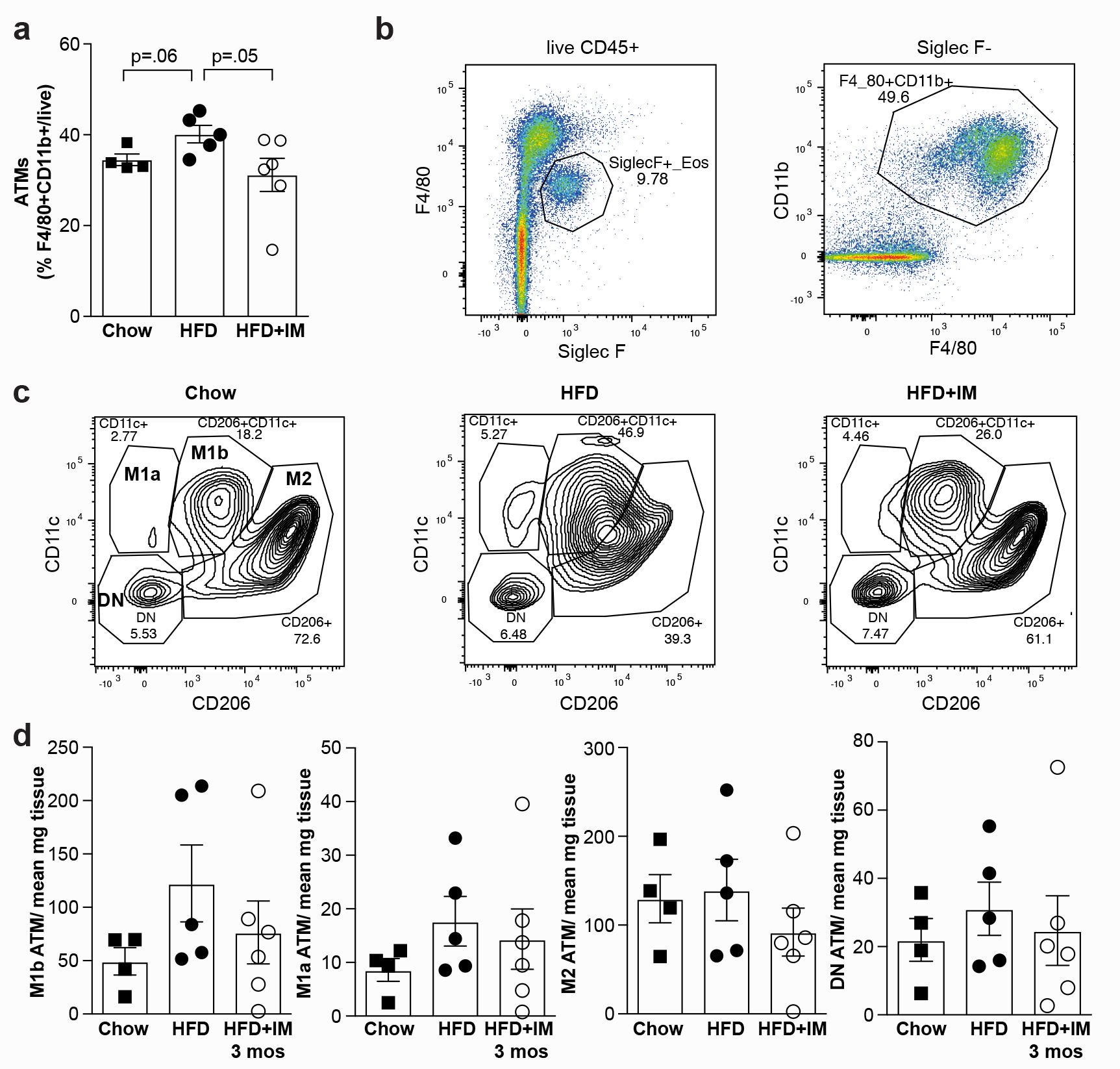
**

**Supplementary Figure 4.** **Adipose tissue macrophages upon imatinib treatment.** (**a**) Quantification of adipose tissue macrophages (ATMs) as percentage of live cells by flow cytometry of chow, HFD and HFD+IM-treated mice (n = 4-6). (**b**) Gating strategy to identify ATMs (single live non-eosinophils CD45+CD11b+F4/80+) by flow cytometry. (**c**) Representative flow cytometry plots for ATM subpopulations (DN: CD11c^-^CD206^-^; monocyte-derived M1a: CD11c^+^CD206^-^; inflammatory M1b: CD11c^+^CD206^mid^; anti-inflammatory M2: CD11c^-to low^CD206^high^). (**d**) Quantification of absolute cell numbers of ATM subpopulations M1a, M1b and M2 in chow, HFD and HFD+IM-treated mice (n = 4-6). ATM: adipose tissue macrophage, HFD: high fat diet, IM: imatinib, DN: double-negative.

**
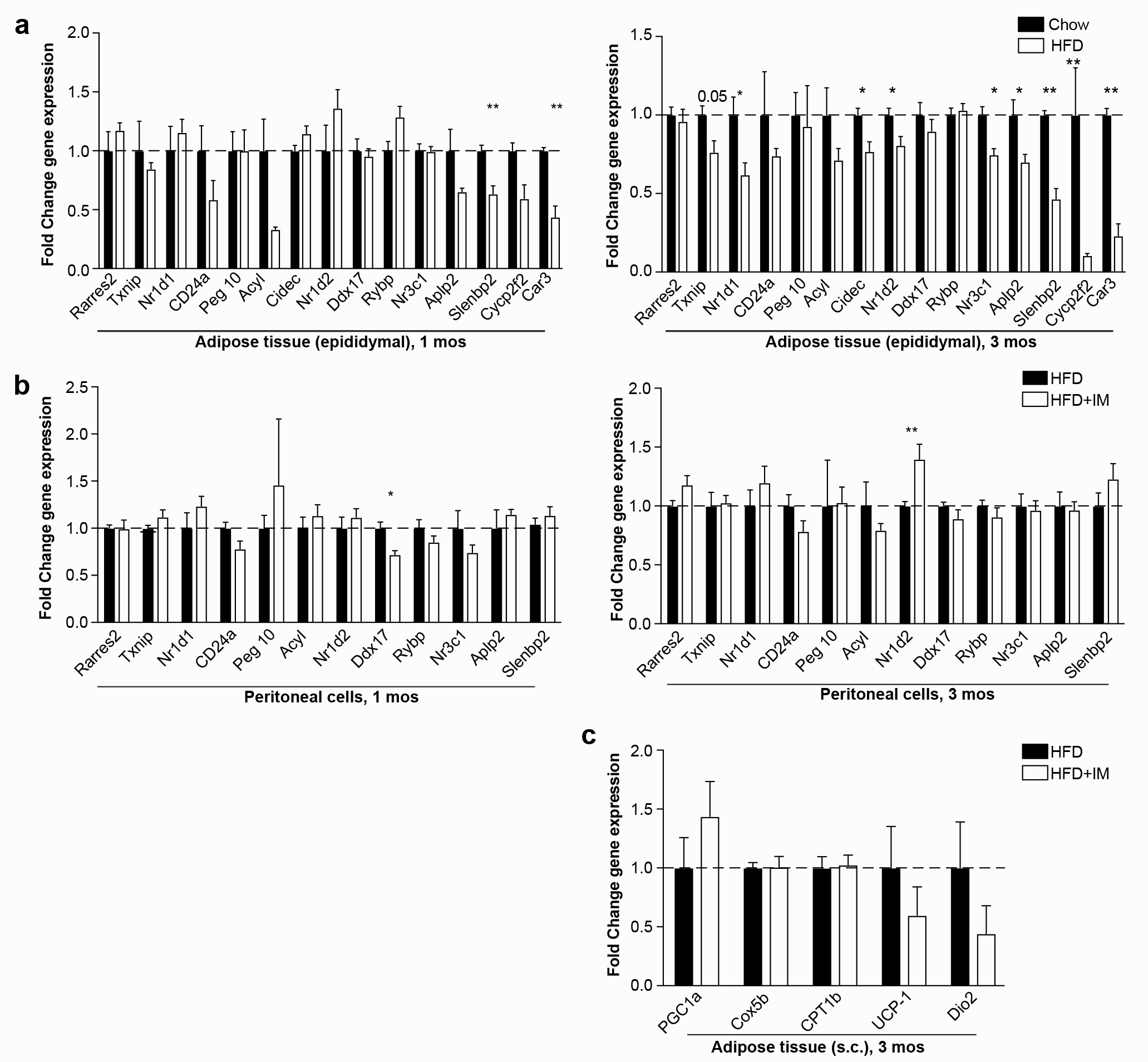
 Supplementary Figure 5.** **PPARγ-phosphorylation-regulated genes in adipose tissue and peritoneal cells.** (**a**) Effect of HFD on PPARγ-phosphorylation-regulated genes in adipose tissue compared to chow after one (left) and three (months) (n=3-9). (**b**) Effect of imatinib on PPARγ-phosphorylation-regulated genes in peritoneal cells in mice on HFD after one (left) and three months of imatinib treatment (right) (n= 5-10). (**c**) Fold change gene expression of genes related to adipose tissue browning after three months of imatinib treatment. HFD: High fat diet, IM: imatinib. Data are presented as mean±SEM. *p<.05, **=p<.01.
